# Value landscapes in interdisciplinary and transdisciplinary research and assessment: exploring indeterminacies and disconnects

**DOI:** 10.1057/s41599-026-06785-0

**Published:** 2026-03-10

**Authors:** Anne-Sophie Schaltegger, Bianca Vienni-Baptista

**Affiliations:** Cultural Studies of Science and Technology Group, Transdisciplinarity Lab, D-USYS, ETH Zürich, Zürich, Switzerland

**Keywords:** Science, technology and society, Social anthropology

## Abstract

Interdisciplinary and transdisciplinary research are promoted because of their contribution to addressing complex societal challenges. However, barriers to these research modes persist, some of which emerge from challenges in assessing inter- and transdisciplinary research. We employ the sensitising concept of ‘values’ to study the entanglement of inter- and transdisciplinary research practices and their assessment. Acknowledging normative and pragmatic approaches to values, we use the metaphor of ‘value landscapes’ to offer a rich perspective on how values permeate research practices and research assessment. Thereby, we seek to contribute to the discussion of values in inter- and transdisciplinarity by offering a perspective on the barriers emerging at the intersection of research practices and research assessment, which have been attributed to gaps in values and valuations. Based on a systematic literature review, we identify two value landscapes in which inter- and transdisciplinary research practices and assessments are embedded: the value landscape around societal relevance, and the value landscape around the research merit of inter- and transdisciplinary research. We outline which values constitute these two value landscapes, and how they relate to each other, as well as to inter- and transdisciplinary research practice and assessment. Two main insights from our study are discussed: (i) the indeterminacy of values around inter- and transdisciplinary research, which has implications for questions around power in their assessment, and (ii) the disconnect that characterises expectations that inter- and transdisciplinarity face, which prompts us to revisit related notions of accountability. We conclude by highlighting the need for new ways of understanding values and their roles in inter- and transdisciplinarity.

## Introduction

The current research environment is marked by policy, societal, and scientific actors alike advocating for inter- and transdisciplinary research (IDR/TDR) due to their potential to contribute to solving complex societal challenges (OECD 2020; Wernli and Ohlmeyer 2023). However, the barriers to IDR/TDR are manifold (Bammer et al. 2020; Donovan 2020). One widely discussed issue is the assessment of IDR/TDR, which often fails to account for the unique characteristics of these modes of research (Laudel and Origgi 2006; Laursen et al. 2022). The term ‘research assessment’ refers here to the evaluation and monitoring of research projects, both *ex ante* and *ex post*, for formative, process, outcome and impact assessment purposes (CDC 2011; Laursen et al. 2023).

The assessment of IDR/TDR poses several challenges. Firstly, it is complicated by different understandings and a lack of definition of what IDR/TDR entails (Vienni-Baptista et al. 2023). Relatedly, there is a lack of clarity about adequate indicators and assessment methods (de Rijcke et al. 2019; Smit and Hessels 2021), resulting in elusive definitions of how interdisciplinary quality is measured in outputs and activities (Lindvig and Hillersdal 2019). Secondly, assessment of IDR/TDR tends to require more time and effort, as inter- and transdisciplinary aspects need to be evaluated in addition to disciplinary ones (Vienni-Baptista et al. 2020). This is also due to the fact that generally, the qualitative approaches that are promoted to evaluate IDR/TDR use more resources and put additional burden on reviewers (Science Europe 2020). Thirdly, IDR/TDR assessment requires skills and expertise that go beyond disciplinary domains, and that include the ability to assess the emergent whole of IDR/TDR (McLeish and Strang 2016). Such experts are difficult to find, as the pool of individuals with fitting epistemic profiles is small, and evaluators are not used to assessing impacts that lie outside traditional conceptualisations of research excellence (Klein 2008; Derrick and Samuel 2017).

These challenges of evaluating IDR/TDR affect related research practices often negatively (Lindvig and Hillersdal 2019). For example, panel members may be biased towards their own disciplines and thus judge inter- and transdisciplinary work less favourably (Lamont et al. 2006). Similarly, common metrics may be biased against IDR/TDR but are attractive to panel members in the light of difficulties in finding indicators (Rafols et al. 2012). It follows that IDR/TDR proposals are disincentivised by current assessment structures and disadvantaged in funding processes (Shapiro 2014; Agate et al. 2020).

Based on a systematic literature review (Jahan et al. 2016), we analyse the values discussed in the interdisciplinary and transdisciplinary academic literatures, and how this study of values is central to better understanding the challenges of research assessment in IDR/TDR. The scholarship on IDR/TDR is spread over different communities of knowledge and with our review, we aim to bridge these dispersed bodies of literature (Vienni-Baptista et al. 2020). The two questions guiding the present study are as follows: What value landscapes emerge from the bodies of literature on inter- and transdisciplinarity? How can an investigation of these value landscapes inform our understanding of the challenges of research assessment and the resulting barriers to IDR/TDR? We argue that it is necessary to develop new ways of studying and understanding the values involved in IDR/TDR processes as well as in their assessment to understand which values are present, how researchers and reviewers enact them, and how they evolve (Dussauge et al. 2015). Doing so will support overcoming the widely lamented gap in the assessment of IDR/TDR (Frickel et al. 2017).

Taking an anthropological stance towards values, we consider both their meaning as cultural phenomena as well as how they relate to social dynamics (Robbins and Sommerschuh 2016). To address both aspects, we acknowledge two theoretical traditions in the study of values: value theory within moral philosophy (Schroeder 2025), and the sociological, pragmatic approach of valuation (Helgesson and Muniesa 2013). Thus, we understand values as normative representations that indicate what artifacts, beings, relationships, and abstract concepts are desirable (Graeber 2001; Elliott 2022). Further, we take values to be enacted through as well as shaped by valuation practices (Helgesson and Muniesa 2013). We use the metaphor of ‘value landscapes’ (Brosch and Sander 2016) to study the values as they manifest in the academic literature on IDR/TDR. As an interpretive lens, the metaphor allows us to understand values as plural phenomena, whose relationships, meanings, and salience vary between contexts and individuals, thus resulting in different configurations (Kaiser 2024).

Values are central to collaborative research and the spaces in which that research happens, in academic as well as policy and funding spaces (Hesjedal 2022; Kaiser 2024). Values lie at the heart of the differences between disciplines and their internal governance of how knowledge is produced, as they determine which research problems are prioritised and how they are framed, which types of knowledge enter the research process, what is accepted as a successful outcome, and how careers are shaped (Piso 2016; Osbeck and Nersessian 2017; Hessels et al. 2019; Klein 2021a; Hammarfelt et al. 2024). Thus, they constrain decisions and actions in scientific research (Robinson et al. 2016). Moreover, values are essential to questions of power and hierarchies (Fritz and Binder 2020), which are central to and can shed light on the assessment of IDR/TDR (Shore and Wright 2000; Huutoniemi and Rafols 2017).

Despite their centrality, empirical and theoretical contributions explicitly addressing values in IDR/TDR and their assessment are scarce, and a cohesive and coherent discourse has not yet been established (Laursen et al. 2021). Often, “values and their role are […] discussed in elusive terms” (Horcea-Milcu et al. 2019, 1425). While it is acknowledged that IDR/TDR and their assessment involve pluralistic values (MacLeod 2018; Laursen et al. 2021), a more in-depth discussion of the concrete meaning of those values and their relation to IDR/TDR practice and assessment is needed. Considering such values explicitly would allow us to critically account for underlying assumptions in inter- and transdisciplinarity (Todt and Luján 2014). We show in this paper that two connected value landscapes underlie discourses in the literature on IDR/TDR: the value landscape around societal relevance, and the one around their merit as modes of research. We outline how IDR and TDR are embedded in these different landscapes and what values guide actions and decisions in IDR/TDR practice and assessment.

The outlined approach to IDR/TDR and their assessment, as well as the role of values in them, is founded on our anthropological backgrounds. With this perspective, our study seeks to contribute to the larger interdisciplinary and transdisciplinary scholarship (Klein 2021b; Darbellay 2024), where interdisciplinarity and transdisciplinarity are themselves objects of research and theorisation (Vienni-Baptista 2024). Conceptually, we also build on and contribute to the field of research evaluation (Lamont 2009; Kulczycki 2023) in the way we understand and disentangle the delineated issues and intricacies of IDR/TDR and their assessment. This study aims to be relevant for funders and assessors concerned with IDR/TDR assessment, as well as researchers practicing IDR/TDR and facing research assessment.

The paper is structured as follows: First, we present the theoretical background on values and IDR/TDR. Then, we introduce the methodological steps of the literature review. After that, the findings focus on two value landscapes emerging from the literature. This leads us to discuss two main insights: (1) the indeterminacy of values in IDR/TDR due to their plurality and context-dependency, and (2) the disconnect observed among values in IDR/TDR, resulting in fragmented expectations that at times stand in tension. The insights are followed by a discussion on the dichotomy of the two presented value landscapes. We conclude with a call to re-position the role of values in IDR/TDR and their assessment.

## Theoretical background

In this section, we present the main concepts guiding our study of values in IDR/TDR practice and research assessment. To start, it is necessary to acknowledge heterogeneity in the terms ‘interdisciplinarity’ and ‘transdisciplinarity’ (Vienni-Baptista et al. 2022), as well as the variety of related practices (Undurraga et al. 2023). We employ the following definitions for the current study: with ‘interdisciplinarity’, we refer to research situations where researchers from different academic disciplines collaborate and integrate perspectives, methods, and/or theories to tackle complex questions, problems or themes that cannot be managed by one discipline alone (Klein 2017). By ‘transdisciplinarity,’ we denote research modes that transcend the academic realm and involve stakeholders in the research process, generally with practical aims (Hirsch Hadorn et al. 2008).

This article treats IDR and TDR together, as they share certain attributes like involving pluralistic values and doing research across boundaries. Nevertheless, when the presentation of our findings requires us to highlight differences between the two, we will refer to IDR or TDR, respectively.

### Values and value landscapes

Anthropologists have discussed the lack of a unified anthropological theory of value(s) (Otto and Willerslev 2013; Steinert 2023). However, relevant accounts have acknowledged the need to conceive of values both in terms of their meaning and function as cultural phenomena, as well as in terms of how they relate to human action and social dynamics (Graeber 2001; Robbins and Sommerschuh 2016; Steinert 2023). Thus, we rest our understanding of the concept of ‘values’, specifically values in science and evaluation, on two epistemic traditions. Firstly, we follow the philosophical value theory in using the concept of values to refer to normative representations that indicate and help define what things (e.g., artifacts, beings, relationships, abstract concepts) are good or desirable (Graeber 2001; Todt and Luján 2014; Robbins and Sommerschuh 2016; Elliott 2022). Secondly, we draw on sociological perspectives on valuation, which state that values may be both enacted through and shaped by valuation practices – social practices that represent and influence what counts as valuable (Lamont 2012; Helgesson and Muniesa 2013). Both approaches have been drawn on by anthropologists discussing a value theory for anthropology (Steinert 2023). We further use the metaphor of ‘value landscapes’ to structure our thinking around values as plural but related, and context-dependent phenomena (Kaiser 2024). In this section, we elaborate on these approaches and their relevance and contribution to the current study.

Acknowledging that philosophical approaches to values are multiple and diverse, we draw on value theory within moral philosophy (Schroeder 2025). Our understanding of the concept of ‘values’ is founded on the philosophical study of values that is concerned with deontic and evaluative, i.e., *normative* dimensions of values, meaning that values indicate what is good or bad, right or wrong (Kaiser 2024). Values are representations that inform about what is considered important or desirable: they represent which things are “matters of concern or care” (Dussauge 2015, 275). As normative reference points, they thus guide how legitimacy and worthiness is judged and determine what can justifiably be demanded (Graeber 2001; Tappolet and Rossi 2016; Elliott 2022). As such, they provide normative orientation in cultural webs of meaning (Robbins and Sommerschuh 2016). We demarcate ‘values’ from ‘value’ in the sense that while ‘values’ are normative representations, ‘value’ describes the *level* of desirability or *worth* of a thing (Graeber 2001; Buchanan 2018). While the two phenomena can occur separately – for example, economic worth does not necessarily imply normative goodness – they can also overlap: something of great value may also be *a* value, or being *a* value may increase its worth (Buchanan 2018).

The philosophical discourse on values has also entered the philosophy of science in ways relevant to our study (Elliott 2022). Here, the distinction between epistemic and non-epistemic values has been prominently discussed. Epistemic – or cognitive – values are values that in some way or another support or promote the success of a scientific endeavour. These are contrasted with non-epistemic values, values that are social or political (Douglas 2009; Elliott 2022). Along with this distinction, one part of the philosophical discourse on values in science has been concerned with the value-free ideal (Douglas 2009). The ideal holds that value judgements which relate to decisions about the legitimacy and acceptability of scientific evidence, scientific conclusions, and their relationship should only ever be based on epistemic values (Douglas 2009; Elliott 2022). As a consequence, non-epistemic values may not play a legitimate role in the ‘internal’ aspects of scholarly knowledge production (Elliott 2022; Longino 2002). The value-free ideal has been contested and called into question on several grounds (Longino 2002; Douglas 2009; Rooney 2017). Nevertheless, it is part of a valuable theoretical discourse that offers a perspective through which to understand the normative representations associated with IDR/TDR, as well as their expected outcomes and contributions as reflected in related evaluative measures in the current study.

To inform our understanding of how values relate to human action and social life, we draw on the pragmatist approach of valuation studies (Dewey 1939), which conceptualises how values are enacted, and thus (re)produced and transmitted by human beings in a specific context (Steinert 2023). The focus lies on valuation as a practice: actions that attribute importance to things (Brosch and Sander 2016). Such attribution happens, e.g., through the investment of resources, or through discourses where worth is expressed and thereby attributed to certain things (Graeber 2001; Hah 2020). Importantly, as valuation practices enact values and thereby indicate what counts as valuable, they also *make* the valued things desirable through that investment (Graeber 2001; Helgesson and Muniesa 2013). Thus, while values represent what matters are of concern or care, valuation practices may indicate as well as produce such matters (Dussauge 2015). From a valuation perspective, values are therefore not taken as stable entities but are made and enacted by different actors (Dussauge et al. 2015). Values emerge not in a vacuum, but in material and social interactions, and so are situated and contextual rather than absolute and universal (Smolka 2022). We acknowledge that the relationship between values in the form of cognitive representations or beliefs and human motivation and behaviour is a contested one (Kaiser 2024), the discussion of which goes beyond the scope of this publication. The valuation approach holds relevance because it foregrounds the generative power of valuation practices in evaluation and assessment.

Evaluation is in itself a form of valuation, in the sense that it attributes worth or importance (or denies said worth) to the evaluated thing, i.e., the evaluand (Davidson 2005; Vatin 2013). Understanding evaluation as a valuation practice further implies it to be a social and cultural process containing a value judgement based on a contextual set of values (Lamont 2012; Vatin 2013). Value judgements, in this context, are evaluative decisions that require the weighing of values or determining to what extent a value has been fulfilled (Elliott 2022).

We further use the metaphor (Goyal and Howlett 2019) of ‘value landscapes’ (Kaiser 2024) to illuminate the plurality and relationality of values in IDR/TDR and their assessment. The metaphor implies that plural values co-exist in different relationships, and that the intensity and pertinence of a value vary between contexts and actors (Brosch and Sander 2016; Kaiser 2024). This means that values as normative representations that serve as reference points for judging something as positive or negative may hold different and varying levels of attraction (positive valence) and repulsion (negative valence) across contexts and actors, whereby attractions have been conceptualised as hills, and repulsions as valleys, resulting in diverse and changing landscapes (Brosch and Sander 2016). Thus, the metaphor allows us to specify our value concept with several key characteristics (Kaiser 2023; Kaiser 2024):

1. Plurality: in any given context, multiple values are at play.
2. Contextuality: the meaning and intensity of a value depend on the context.
3. Relationality: values stand in relation to one another; other values are part of the context that influences the meaning and salience of a value. These relationships are dynamic and can be hierarchical in nature (Schulz et al. 2017).

Different compositions of values in different relationships in different contexts thus result in different *value landscapes*. The composition of a value landscape at a given time, held by, e.g., a specific individual, will then influence said individual’s valuation practice at that time based on which value(s) have the highest intensity, i.e., attraction and thus salience (Brosch and Sander 2016). We see the normative dimension of values, considering something as ‘good’ or ‘bad’, to match the idea of attractions and repulsions stated above, and the metaphor’s implication of changing contexts and relationships to allow for a view on values as made, enacted, and dynamic. Thus, we use the metaphor as an interpretive guide in our grounded theory approach to values as suggested by Kaiser (2024), in contrast to studies applying the metaphor as a theoretical framework in quantitative research (Bremer et al. 2014; Schulz et al. 2017).

### Values in interdisciplinary and transdisciplinary research and assessment

In discussions of values in IDR/TDR, the most prominent topic has been that of value pluralism (Laursen et al. 2021), which emerges when researchers from different disciplines with distinct value landscapes collaborate (MacLeod 2018). Studies show that value pluralism in IDR poses a challenge, creating a demand for facilitative interventions and training to foster the understanding and skills needed to navigate values across disciplines and societal spheres (O’Rourke and Crowley 2013; Caniglia et al. 2023). As a means to manage such pluralism, Laursen, Gonnerman, and Crowley (2021) propose philosophical dialogue interventions to tackle the overlooked challenge of value-related conflicts. Koskinen and Rolin (2022) further acknowledge the required context-sensitivity when discussing values in TDR, especially when it comes to deciding which values may play a legitimate role in transdisciplinary knowledge production.

Emotions are important markers of value differences between disciplines (Salmela and Mäki 2017; Kaiser 2024) and constitute a highly relevant aspect of IDR/TDR and related enactments of values by showing where differences emerge and where spaces for critical reflection should be opened up (Boix Mansilla et al. 2016; Smolka et al. 2021). Emotions emerging in IDR contexts have been described as discomforting or unsettling, such as frustration over knowledge gaps in oneself or others that complicate collaborations, or doubt and mistrust in another discipline’s methodology (Fitzgerald et al. 2014). However, Hesjedal (2022) also stresses the importance of positive affective processes that support the success of IDR/TDR by filling collaborative spaces with meaning and values, thus actively shaping it as a social ‘place’. Positive or negative affective reactions may also arise when researchers are subject to assessment: Robinson-Garcia et al. (2023) show how researchers in different fields are frustrated by the need to balance different valuation regimes in their work, especially due to the experienced conflict between their own values and those used to assess them. Research assessment in IDR/TDR can further lead to frustration if the recognised or required outputs become too broad, overwhelming researchers, or if the breadth of the assessment disregards disciplinary specificities (Muhonen and Himanen 2025).

In a study among members of the American Evaluation Association, which represent both evaluators across different fields (research evaluation, but also personnel evaluation, programme evaluation, etc.) as well as researchers from different disciplines, Wanzer (2021) has shown that researchers and evaluators agree that value judgements in the form of attributing merit or value to an evaluand are a central goal of any kind of evaluation. Examples of such value judgements could be decisions of whether enough efforts have been shown to make scientific outputs publicly accessible – based on the value of open science – or whether consumer needs have been adequately met – based on the value of, e.g., transferability of knowledge (Davidson 2005; Elliott 2022). Thus, values need to be taken into account and value differences can occur – in assessment panels, diverging valuations of projects can lead to value conflicts, and emotions regulate negotiations between panellists (Brunet and Müller 2023). Such differences are more pronounced in IDR/TDR assessment panels, where the panels themselves are diversely constituted (Klein 2008). Given also the perceived differences between values of scholars and values in academic assessment (Robinson-Garcia et al. 2023), Agate et al. (2020) argue for aligning the two. This is especially relevant in the case of IDR/TDR, where value conflicts emerge from assessment mechanisms that foster competitiveness and individual success, and restrict time resources. Thereby, interdisciplinary and transdisciplinary quality and impact are not incentivised, as more challenging and time-consuming collaborations between disciplines and with stakeholders are inhibited (Agate et al. 2020). Felt (2017) highlights that temporal orders in academic assessment reinforce detrimental effects for “collective knowing and more integrative thinking” (p. 62), as research resulting in outputs or impacts that do not align with imposed timelines is not favoured by the governing system. Felt (2017) poses the question of whether current assessments foster the type of academic values the scientific community would agree upon, and how academic values and assessment indicators can be brought into accord. Including both scholars and non-academic stakeholders involved in the research process in the development of assessment criteria has been one suggestion to tackle the alignment of these differing values (O’Connor et al. 2019; Belcher et al. 2020).

## Methods

This study is based on a systematic literature review (Jahan et al. 2016) conducted in the research project “Investigating interdisciplinarity and transdisciplinarity: Intersections of practices, culture(s) and policy in collaborative knowledge production” (INTERSECTIONS)^1^. Guided by the PRISMA protocol (Page et al. 2021, Fig. 1), the literature review aimed to disentangle four themes related to the INTERSECTION project’s main aims: (i) understandings of interdisciplinarity and transdisciplinarity; (ii) theories and methods in IDR/TDR; (iii) challenges to IDR/TDR; and (iv) intersections between practices, cultures, and policy in collaborative settings (Vienni-Baptista 2024).

**Fig. 1:**
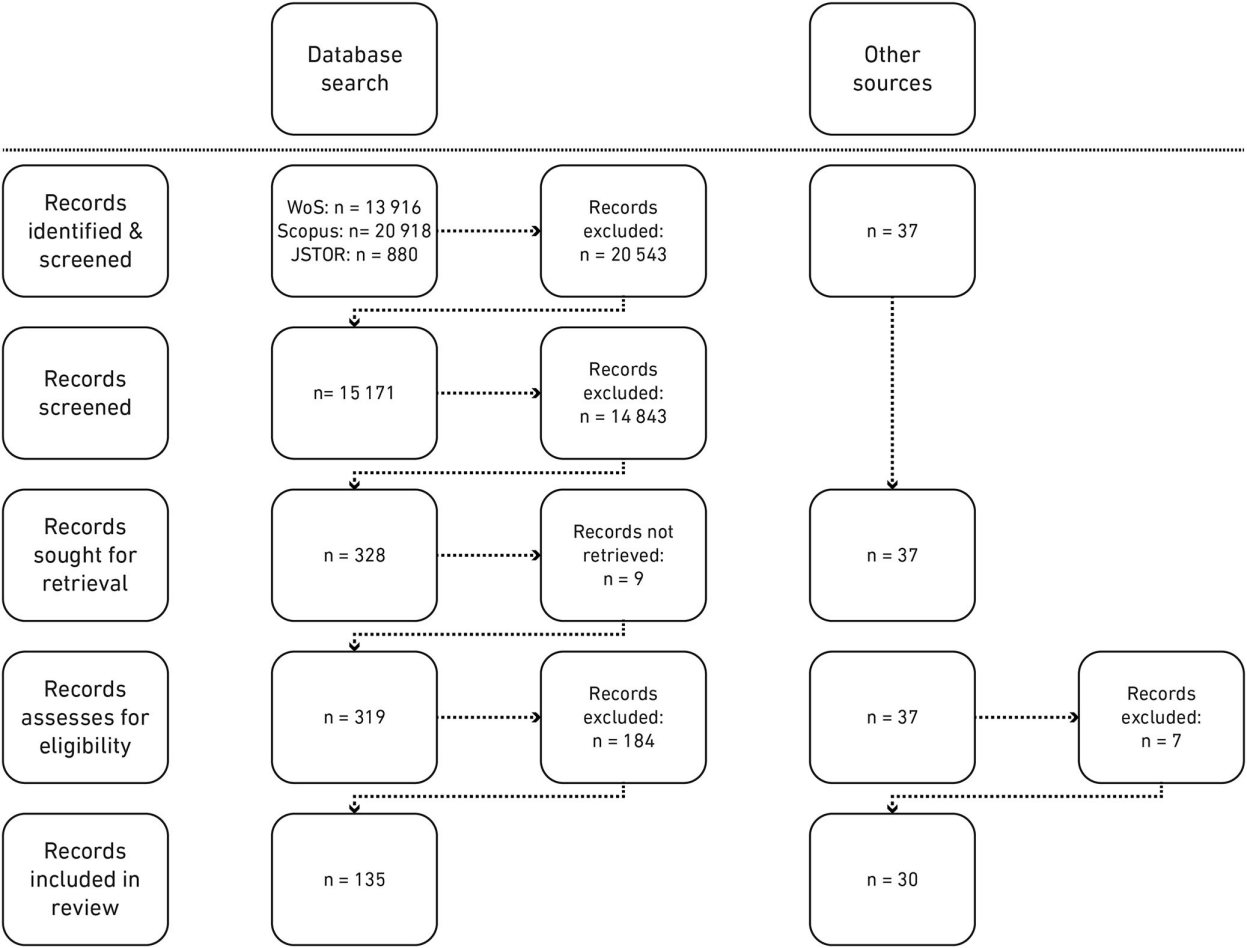
Overview of the search and selection process after PRISMA (Page et al. 2021).

**Fig. 2:**
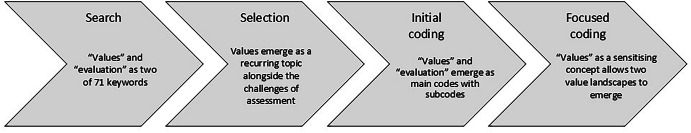
Overview of the emergence of the concepts of ‘values’ and ‘assessment’ throughout the literature review.

As recurring themes in the initial exploration of the literature, ‘values’ and ‘evaluation’ were defined as two of 71 keywords to guide the systematic literature search. Only during the initial coding, their importance and especially their relationship started to emerge as a promising theoretical avenue to offer new insights, which was further established and explored during the focused coding process. Figure 2 shows an overview of how the concepts of values and assessment emerged and were considered in the different steps of the systematic literature review.

### Data collection

Based on an initial exploratory scanning of relevant publications in the interdisciplinary and transdisciplinary literature, three team members operationalised the four thematic foci by jointly defining sets of keywords (Table 1). These keywords captured the main concepts and discourses distilled from this initial exploration and were designed in a broad way that would allow less well-known literature to emerge alongside established and well-known contributions. These were assembled to form complex search strings (Schaltegger and Vienni-Baptista 2023) used to search Web of Science (WoS), Scopus, and JSTOR for the period of 2000–2023. In pooling these three sources, we achieved broad coverage of journals and related topics (Mongeon and Paul-Hus 2016; Singh et al. 2021). Databases resulting from the literature search were merged, and 20,543 duplicates were removed^2^, resulting in 15,171 records.

**Table 1:** Keywords for the search query.

Set A	Set B	Set C	Set D	Set E
Interdisciplinarity/ Transdisciplinarity	Understanding	Intersections	Cultures	Research
interdisciplinar*	understand*	relat*	discipl*	research*
transdisciplinar*	defin*	intersect*	knowledge*	practi*
	theor*	gap*	epistem*	process*
	concept*	partner*	cultur*	problem*
	mode	transfer*	power*	competenc*
	principle*	connect*	identit*	scien*
	modalit*	cross*	valu*	co-produc*
		boundar*	creat*	coproduc*
		integrat*	communit*	collabor*
		space	communic*	participa*
				art
				perform*

Set F	Set G	Set H	Set I	
Policy	Methods	Challenges	Education	
polic*	method*	success*	teach*	
politic*	stud*	fail*	student*	
fund*	investigat*	complex*	educat*	
recommend*	approach*	factor*	pedagog*	
govern*	framework*	condition*		
impact*	tool*	challeng*		
stakeholder*	devic*	barrier*		
citizen*		indicat*		
PPP				
institut*				
program*				

The list of records underwent manual cleaning based on title and abstract, guided by a set of collaboratively developed inclusion and exclusion criteria (Jahan et al. 2016) (see supplementary file). The team then selected 319 publications to be considered for coding, together with 37 records that were manually added (Schaltegger et al. 2023). The resulting set of literature was spread across different fields and communities of knowledge, namely, evaluation and policy studies, philosophy of science, integration and implementation science, science and technology studies, innovation studies, sustainability science, and the scholarship of interdisciplinarity and transdisciplinarity. This represents the fragmentation of the literature on IDR/TDR (Vienni-Baptista et al. 2020), which we aim to bridge with this literature review.

### Data analysis

For the data analysis, three team members simultaneously coded 165 out of the 356 selected records. The 356 publications had been categorised according to which of the four thematic foci they potentially contribute to, based on titles and abstracts. All publications that were categorised into more than one thematic focus were coded first. The remaining publications were prioritised based on an estimation of whether they introduced new aspects. The project team held weekly meetings to adjust the coding process until theoretical saturation was reached for each thematic focus, meaning that the structure of the codebook stabilised and no new codes or subcodes needed to be established (Charmaz 2014; Hennink et al. 2017; Saunders et al. 2018). Disagreements were discussed until a shared understanding was established, and the collaborative coding process was accompanied by consistent memo-writing about each team member’s observations and collective decisions (Charmaz 2017).

In this first phase, we applied initial coding to inductively develop the codebook, allowing for themes and salient concepts to emerge from the literature. During this step, the codebook was established, and continuously refined and restructured in an inductive manner without any preexisting, deductive categories, paying close attention to the data and its contribution to our understanding of the four thematic foci (Charmaz 2014). Codes were thus interpretive categories describing the relationship between the data and our thematic foci. ‘Values’ emerged as a main code, with subcodes representing two dimensions of the concept identified in the literature: (i) types of values and (ii) roles of values in the research process. ‘Evaluation’ emerged as a code pertaining to different challenges and barriers to IDR/TDR in the assessment process, thus highly relevant to the third thematic focus of the overall literature review. We then used the term ‘assessment’ to encompass both evaluation and monitoring (Laursen et al. 2023).

In the second phase, focused coding was used to condense and sharpen the results from the first phase, specifically for the current study (Charmaz 2014). The initial coding implied the important but understudied role of values in IDR/TDR and suggested values as a theoretical avenue to better understand the challenge current research assessment poses for IDR/TDR. By employing ‘values’ as a sensitising concept that provided guidance for research and analysis without being prescriptive (Blumer 1954), we synthesised the larger corpus of data along the research questions guiding this paper. Thereby, we “advance[d] the theoretical direction” (Charmaz 2014, 138) of establishing our understanding of how values in IDR/TDR, as seen through the literature, relate to and influence the challenges of assessing IDR/TDR practices and contributions. Thus, during the focused coding, new codes emerged that focused on the nature and role of values in IDR/TDR and their assessment, some examples of which are presented in Table 2.

**Table 2:** Extract from the focused coding codebook on ‘values’.

List of Codes	
*Relevance and social alignment is becoming much more important*	
Tension with excellence and rigour	
*Tension between collaboration and accountability*	
*Requirements for TD*	
TD needs to be credible and legitimate	
IDTD must adhere to scientific values (‘scientificity’)	
*Evaluation is value-laden*	
Evaluation needs to consider the values of relevance	
Considering values improves evaluation	
Values determine ideas of quality	
Management logic of quality	
Quality often determined scientifically/disciplinary	
Tailoring evaluation to project’s values & goals	
*Identifying values/make them explicit*	
Transparency increases legitimacy and credibility	
to distinguish between acceptable and non-acceptable values	
to increase accountability, because research is not value-free	
to enhance collaboration	
*Making (implicit) value judgements by defining impact*	
*Involvement of values (not) recognised*	
Applied problems require weighing of societal value(s)	
Engaging of values by involved individuals	
Societal values enter through stakeholders	
*(Some) values are integral to IDTD*	
TD requires values from practice	
Understanding values to understand TD	
*Societal values and knowledge ground scientific knowledge*	
Values of stakeholders lead to social robustness	
Value judgements necessary to guide scientific decisions	
Societal values have to be interpreted	
Stakeholder involvement increases accountability and relevance	
*‘The value’ of IDTD*	
Needs to result in scientific and societal progress	
Usability of knowledge in policy	
TD: entering of societal values	
Creating a more comprehensive picture of reality	
IDTD produce solution for problems	
Social robustness through IDTD	
Societal compatibility of knowledge	
*Value pluralism*	
Value pluralism creates conflict (constructive or destructive)	
due to	
academic vs. societal values	
Complex problems characterised by value pluralism	
Different disciplines have different values	
Dealing with value pluralism	
Self-awareness and reflexivity	
Mismatch between funders’ and project’s values	
Top-down values govern research	
Structures don’t promote values of TD	
*Values*	
Relevance vs. excellence	
Usability	
Reliability	
Competition vs. collaboration	

Sensitising concepts only have vaguely defined properties which do not restrict questions and analytical avenues to be pursued (Blumer 1954; Charmaz 2014): they guide attention and support the development of ideas (Bowen 2006). We did not follow an operationalised understanding of what values are or entail in IDR/TDR. Rather, the analysis was guided by the understanding of values as normative representations that indicate matters of concern or care (Dussauge et al. 2015). Thus, matters that were stated to be important, valuable, or desirable in relation to IDR/TDR were taken to indicate values.

Through the focused coding approach, we identified two value landscapes underlying the discourses in the literature on IDR/TDR and their assessment: a) the value landscape around the societal relevance of IDR/TDR, and b) the value landscape around the research merit of IDR/TDR. The first illustrates that the inclusion of societal and stakeholder values leads to more contextualised and grounded, and hence *relevant* research. The second outlines values that IDR/TDR are expected to live up to in order to be scholarly meritable, the possibility of which is often critically questioned, while showing which values enable IDR/TDR to make unique epistemic contributions at the same time.

## Findings

The findings are structured under the two value landscapes we identified: (a) around the societal relevance of IDR/TDR and (b) around the research merit of IDR/TDR. They influence one another and are tightly enmeshed. In each case, we present values that have been described in the literature, how they relate to IDR/TDR and its assessment, and how they relate to one another, creating a value landscape.

### The value landscape around societal relevance of IDR/TDR

**Fig. 3:**
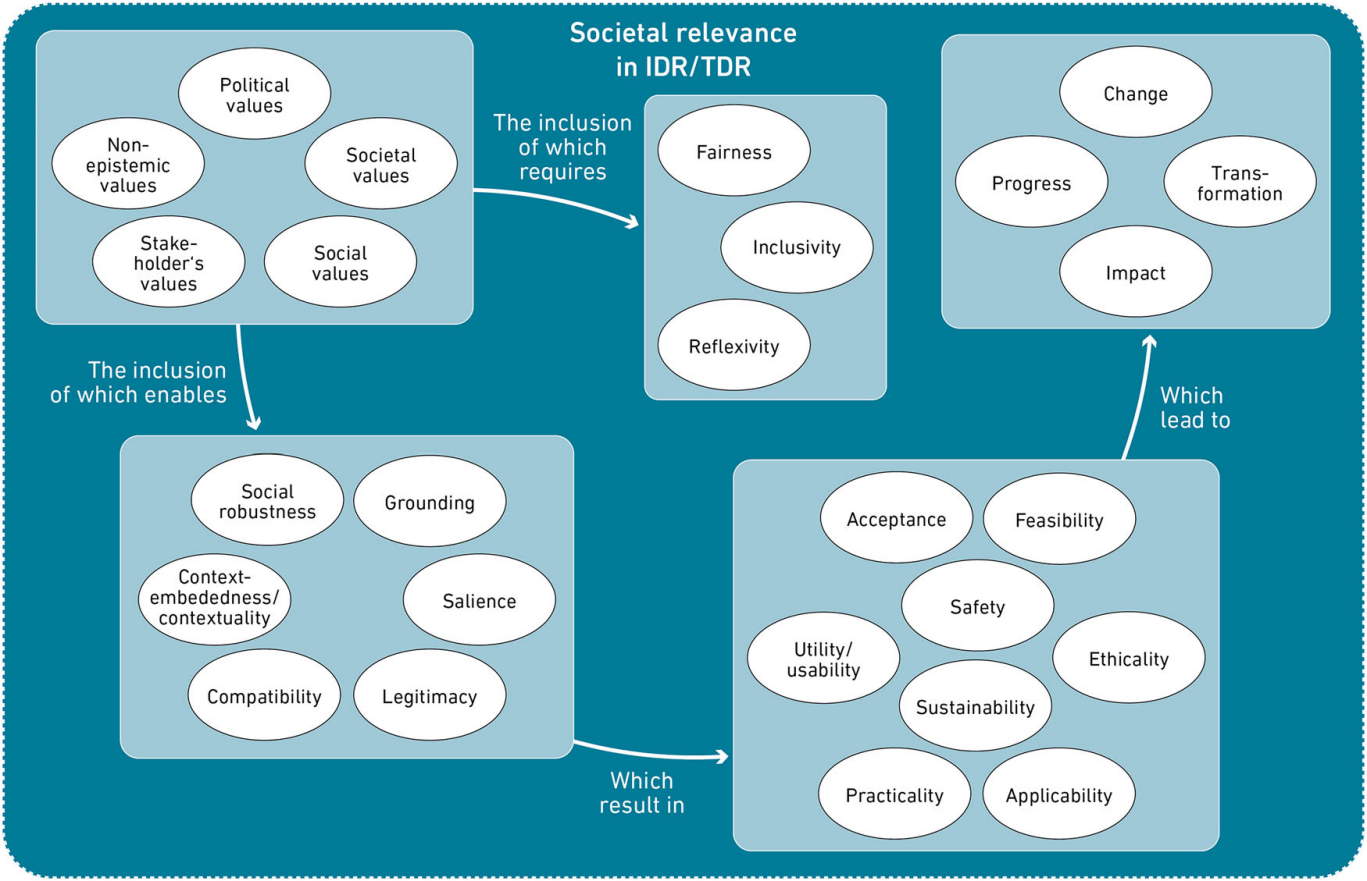
Value landscape around societal relevance. The value landscape around societal relevance consists of several groups of values that are interdependent.

A first value landscape in the literature relates to the promise that inter- and transdisciplinary research produce *societally relevant*^3^ knowledge (Fig. 3).

In the literature on IDR/TDR, societal relevance is conceptualised as having impact on societal spheres (de Sandes-Guimarães et al. 2022), effectiveness in a real-world context (Hirsch and Luzadis 2013), making a contribution to problem-solving (Parthey 2022), or as a consequence of putting contextual knowledge to practical use (Jacobi et al. 2020; de Jong and Muhonen 2020).

The literature shows consensus in that IDR is better equipped to produce societally relevant outcomes because of the complexity of societal issues, which require the contributions and integration of different disciplinary viewpoints to be grasped and addressed in their entirety (National Academies of Sciences et al. 2004; Jacob 2015). Thus, while not a prerequisite, it results from our literature review that IDR is often relied upon to produce societally relevant knowledge and thereby contribute to tackling societal challenges (Lélé and Norgaard 2005; Jacob 2015). The promise of societal relevance is a main reason for funders and governments to push for IDR (as well as TDR) as the answer to the demand for more research that is attuned to societal needs and problems (Felt 2010; Huutoniemi 2014; Bark et al. 2016). Consequently, societal relevance is often seen not only as a result of IDR/TDR but as an inherently defining characteristic and value (Schmidt and Siune 2008; Klein 2014). The production of societally relevant knowledge in IDR is considered to depend on – among other things – the inclusion of what is referred to as *non-epistemic values* (Robinson et al. 2016; Osbeck and Nersessian 2017), *social* or *societal values* (Fazey et al. 2018; Horcea-Milcu et al. 2019), or *political values* (Maas et al. 2022), hereafter subsumed under the term ‘societal values’. The uncertain and intricate nature of the tackled problems requires not only integrating epistemological dimensions but also weighing social values to prioritise between outcomes, benefits, beneficiaries and interests (Lélé and Norgaard 2005; Frodeman 2014).

Like interdisciplinary teams, transdisciplinary teams are equally required to link epistemic-technical and social-political dimensions and incorporate different value landscapes (Krohn 2008; Fisher et al. 2015; Fazey et al. 2018; Maas et al. 2022). However, in TDR specifically, the consideration of societal values is additionally intricate due to the involvement of stakeholders such as community members, citizens, industry representatives, practitioners in TDR in sustainability topics (Boyd et al. 2015; Rodríguez-Labajos et al. 2021; Schneider et al. 2021), and patients in public health research (Groot et al. 2022). Their involvement makes way for case-specific, contextual values to shape the research process (Polk 2015). Both publicly shared, societal imaginaries, as well as project-internal ideas about outputs, impacts, and the future in relation to the tackled problem, are sources of societal values in the TDR process (Felt et al. 2016). Brigstocke et al. (2017) argue that, especially in the TDR process, such social and political values that stem from the inclusion of stakeholders should also be considered in assessment procedures set up by funders, to ensure the relevance of the research. Moreover, there is a recursive element to considering stakeholder’s values: once stakeholders become involved, ‘relevance’ is not just a result but a requirement that ensures stakeholders spend their time in ways that are beneficial to them (Bandola-Gill et al. 2022).

Involving societal values in the research process is not only considered to be a practice that ensures societal relevance but also contributes to the enactment of other values related to this value landscape. We start with the values of *contextuality* and *grounding*, which imply that knowledge which is grounded in a specific reality and is contextually embedded has increased value (Nowotny et al. 2002; LaFave and Dunn 2019). Considering the societal values of a wider community, as discussed in the previous paragraph, is taken to increase the context-embeddedness and ‘groundedness’ of the research (Adelle et al. 2020), and can thus be framed as a valuation practice. Another often-mentioned value with a similar meaning is that of *social robustness*: through engagement of stakeholders in the research process, knowledge production must withstand the stakeholders’ scrutiny and is constantly contested and challenged from various scientific and societal perspectives (McGregor and Donnelly 2014; Engels and Walz 2018). Social robustness is connected to similar values like those of *salience* and *legitimacy* (Belcher et al. 2016). Authors generally make reference to salience and legitimacy as defined by Cash et al. (e.g., 2002; 2003) in a triad alongside the value of credibility. As credibility refers to scientific plausibility and trustworthiness, we will discuss it in the next section as part of the value landscape of research merit. Salience refers to the practical, contextual relevance of the produced knowledge, while legitimacy addresses a knowledge production process as being *fair* and *inclusive* of the heterogeneous values and beliefs of stakeholders (Defila and Di Giulio 2024). This highlights the necessity of understanding values like groundedness or contextuality in relation to other values like fairness and inclusion: only if the selection of considered societal interests was unbiased and diverse and also acknowledged divergent viewpoints, legitimacy and social robustness can be ensured (Defila and Di Giulio 2024). Thus, an isolated perspective on contextuality is not sufficient to ensure that the knowledge produced by IDR/TDR has the promised value: rather, it is necessary to take into account the wider value landscape.

Scholars have taken these values and turned them into principles and indicators of assessment frameworks: Belcher et al. (2016), for example, propose legitimacy as one of four core principles in TDR assessment, alongside relevance itself. Importantly, legitimacy is not taken as a measurable characteristic of the output or outcome of the research, but rather as a value that is represented and ensured by specific practices such as considering the values and interests of affected stakeholders, and setting up transparent and accessible processes (Belcher et al. 2016; Belcher and Claus 2025).

Knowledge and other outputs that are contextually grounded, thus socially robust, legitimate, and salient, are considered to increase the *acceptability* of IDR/TDR results and solutions (Sonetti et al. 2020; Klein 2021a). That is, because socially robust knowledge is considered more likely to adhere to values of *sustainability* and social, economic, and technological *feasibility* in relation to solutions. Moreover, it is ultimately more *useful* (relevant for an application), *usable* (not only relevant, but in its form ready to be used) and *applicable*, as it is produced with the users’ interests and future applications in mind (Balsiger 2004; Henze 2021; Bandola-Gill et al. 2022). Here as well, the values of usefulness, practicality and applicability of knowledge, or more concretely, measurements of the actual utilisation of knowledge, have been included in frameworks for assessment of IDR/TDR (Jacobi et al. 2020; Langfeldt et al. 2020). Maasen and Lieven (2006) further assume that outcomes that are useful due to the consideration of societal values and diverse needs of affected stakeholders are consequently also *safer* and more *ethical*.

Knowledge that is useful and applicable is valued as it can be transferred and therefore become directly conducive to real-life, practical *societal change* and *transformation*, i.e. *impact* – themselves values governing discussions around IDR/TDR in the literature (de Jong and Muhonen 2020; Bergmann et al. 2021). This, in turn, leads us back to definitions of IDR and TDR themselves: while diverse and heterogeneous understandings of ‘interdisciplinarity’ and ‘transdisciplinarity’ exist, some definitions characterise them as research modes aiming to contribute to solving societal challenges. In these understandings, societal impact, change and transformation do represent ultimate goals of IDR/TDR.

The production of societally valuable knowledge is described as a positive, motivating factor, but also as a challenging one (Laursen et al. 2021), due to the values involved in actual IDR/TDR practice being heterogeneous. In the case of IDR, problem formulation and research design require navigating divergent values between disciplines (Piso 2016; Robinson et al. 2016). Researchers with different disciplinary backgrounds have different ideas of what is ‘right and wrong’ (Balsiger 2004). Such differences stemming from, for example, explanatory preferences or standards of evidence create a need for reconciliation and genuine integration between them (Osbeck and Nersessian 2017; MacLeod 2018). Integration, a central goal of IDR (Defila and Di Giulio 2015), thus not only relates to knowledge, but also to values (Freeth and Caniglia 2020), and becomes more challenging if breadth is held as a value and quality indicator of IDR and hence maximised, as suggested by Huutoniemi & Rafols (2017) and discussed in the next section. As a result, *reflexivity* emerges as a central value relating to the practice of interdisciplinary team collaboration. Reflexivity is considered to make teams aware of the values they engage with, enabling them to articulate these values (O’Rourke 2017). Successfully navigating these plural values may also positively affect the outcome of the assessment, as panel members assessing IDR look for well-balanced work that manages to navigate conflicting values (Boix Mansilla 2006).

In the case of TDR, as well, the literature suggests valuing reflexivity and openly discussing values, interests, and priorities to establish common ground (Blanchard and Vanderlinden 2010; Bergmann et al. 2021). Working with stakeholders’ values is not a straightforward process, as different actors might disagree about priorities (Felt et al. 2016). TDR processes thus require negotiations regarding which social and political values are acceptable in what roles in the research process (Koskinen and Rolin 2022). This becomes especially important when co-determining with stakeholders the intended impact, which warrants that implicit value judgements be made explicit (Brigstocke et al. 2017; Howarth et al. 2022). Values relating to successful IDR/TDR processes, however, go beyond reflexivity. Such collaborations require knowledge exchange and communication to be understandable across disciplines and to all non-academic participants (Callaos and Horne 2013; Jacobi et al. 2020). Again, inclusivity is named as an important value for both IDR and TDR, enabling joint responsibility-taking (Bayne-Smith et al. 2008; Polk 2015).

Outlining the values around societal relevance (Fig. 3) and framing them as constituents of a value landscape shifts our attention to the context-dependency of their meaning. Hakansta and Jacob (2016), for example, demonstrate how an understanding of relevance depends on all actors codetermining it. The practical meaning of relevance is therefore dependent on the underlying values coming from the surrounding societal context, the field of research, and the intended audience (Lélé and Norgaard 2005; Flinders 2013). Cash et al. (2003) make a similar argument for the concepts of salience and legitimacy, showing how they are interpreted differently by different actors, depending on the values these actors hold. Striving for societal relevance is in itself a valuation of a specific type of knowledge that is contextually grounded and socially robust. But determining in each case what exactly ‘relevant’ means requires considering different needs and interests, valuing some over others, thus revealing yet another valuation practice. Individuals or institutions deciding what is ‘societally relevant’ therefore exercise a form of power, affecting the research by influencing the value landscape it is part of (Lélé and Norgaard 2005).

### The value landscape around the research merit of IDR/TDR

**Fig. 4:**
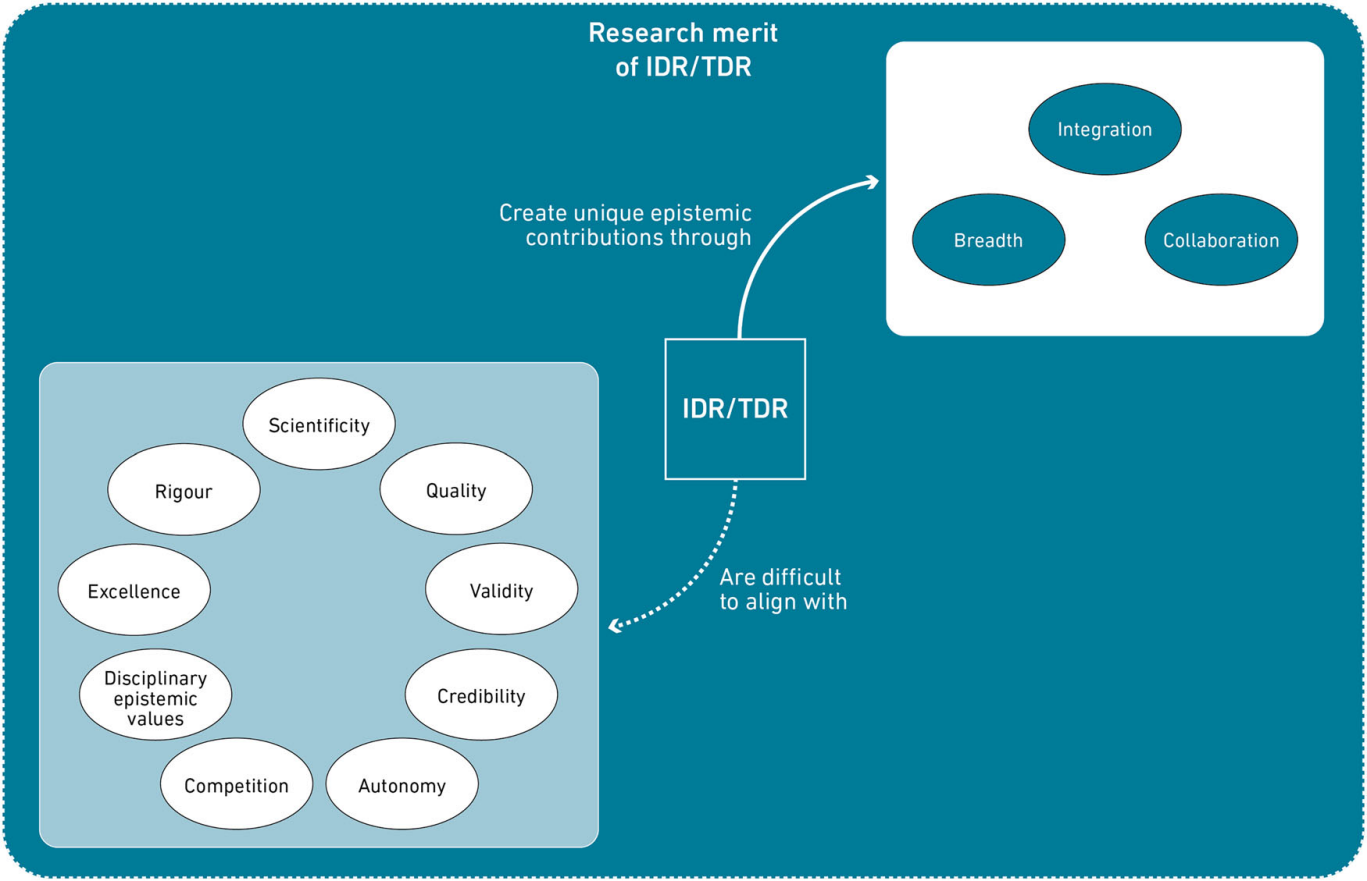
Value landscape around the research merit of IDR/TDR. The value landscape around the research of IDR/TDR consists of two distinct groups of values, of which one is seen as difficult to align with IDR/TDR, while the other is considered to be conducive for the epistemic contributions of IDR/TDR.

In our study, a second landscape materialised, which we label as that of ‘research merit’ (Fig. 4), relating to the worth of IDR/TDR as a form of scholarly knowledge production (Davidson 2005). It is shaped, firstly, by values determining the *Wissenschaftlichkeit* (Henze 2021) (‘*scientificity*’) of IDR/TDR, meaning the level to which standards and norms of academic, scholarly knowledge production are complied with. We use ‘scientific’ here in the German sense of *Wissenschaft*, relating to any systematic scholarship, crucially not just the natural sciences but also the social sciences and humanities (Dahler-Larsen 2006). Secondly, the landscape of research merit relates to values that represent what the epistemic contributions of IDR/TDR are considered to be in comparison to other forms of research considered more traditional (Balsiger 2004).

One aspect of the value landscape revolving around the research merit of IDR/TDR is that they are generally expected to align with standards underlying the two connected values of *rigour* and *validity*. These values are strongly embedded in the academic, disciplinary institutional context in which IDR/TDR operate and depend on, even though IDR/TDR themselves might challenge boundaries between disciplines and between the academic and societal realms (Mäki 2013; Jahn et al. 2019). However, oftentimes, it is questioned whether IDR/TDR can align with these values. One reason for this is that, from a disciplinary perspective, interdisciplinary work is often judged as less rigorous and valid than purely disciplinary research. Callard & Fitzgerald (2015) describe an example of that where the collaboration of Callard as a humanist with a neurologist was considered not to be able to compete with pure neurological work in terms of rigour and being “cutting-edge” (37) due to its interdisciplinary nature. Such concerns can also be attributed to the third value in Cash et al.’s (2002) triad already mentioned in the previous section, that of *credibility*, which refers to the scientific or disciplinary adequacy of the research and is often included as a criterion in frameworks developed for the assessment of TDR (Belcher et al. 2016; Staples et al. 2021). Another reason for questioning the validity and rigour of IDR/TDR is that rigorous research is considered to require well-formed problems, which is difficult to reconcile with the complex or wicked challenges IDR/TDR are concerned with (Huutoniemi 2014). An additional threat to rigour in IDR/TDR is considered to be the necessity to adapt the level of detail and precision in communication across disciplines, to cater to diverse levels of expertise (Callaos and Horne 2013). Lastly, these values are connected to methodological concerns. IDR/TDR need to ensure the rigour and validity of methods of integration, which are, however, often undefined and underreported, thus impeding critical examination (Defila and Di Giulio 2015).

Relatedly, it is a recurring theme that research merit might be compromised by an increased orientation towards societal relevance. While Jahn et al. (2012) present a framework of TDR with which they aim to show that both epistemic and societal progress are possible, they acknowledge that one might be prioritised over the other, depending on the research context. In her study on different types of knowledge production practices in the intersection with policy, Bandola-Gill (2019) not only illustrates this tension but also shows that the *excellence* of research practices or the resulting knowledge is sometimes called into question merely due to its applicability or usefulness to a real-life, contextual problem. Felt et al. (2016) also describe the fear of losing credibility in the academic system, which is especially strong for TDR, and the balancing act researchers make when translating TDR results for an academic audience. Describing how they try to spin a contextually relevant problem or result into a scientifically interesting one implies that these are not congruent. Part of this academic audience are actors involved in research assessment, where scepticism about the compatibility of excellence or rigour with problem-oriented research is oftentimes present and the prioritisation of one value over the other results in their enactment in the form of an evaluative decision (Derrick and Samuel 2016). These examples show that values around societal relevance coexist alongside values of research merit, the latter of which determine the legitimacy of the research and its results in both research practice and assessment. The relationship between values around societal relevance and those around research merit is not evident: while doubts about their compatibility seem to be pervasive, funding institutions increasingly push for and assess both, and panel members see a variety of ways in which they depend on one another (Derrick and Samuel 2016; Bandola-Gill 2019).

Another prominent value is that of *quality*. As with the other values presented in this section, its meaning is dependent on the disciplinary context. Thus, ‘quality’ and connected values themselves exhibit pluralism, as researchers from different fields have distinct epistemic values, which determine what is considered to be an epistemic contribution (MacLeod 2018; de Sandes-Guimarães et al. 2022). Consequently, whether IDR/TDR are deemed valid or acceptable in assessment procedures is based on differing disciplinary standards (Mansilla et al. 2006; König and Gorman 2017) leading to “fractionated definitions and metrics of what constitutes quality” (Feller 2006, 8). Actual meanings of the notion of research quality that hold across disciplines have not been established (Ochsner et al. 2017). Due to this pluralism, assessments of IDR and TDR need to incorporate variable indicators and criteria (Klein 2008).

In contrast to the narrative about the potential shortcomings of IDR/TDR in terms of their scientificity, IDR/TDR are considered to be driven by a set of values that relate to their distinct epistemic benefits and contributions in comparison to purely disciplinary research approaches (Huutoniemi and Rafols 2017). One major such value is that of *integration*. Integration was mentioned in the previous section as a practice to deal with the pluralistic value landscape around societal relevance. For IDR/TDR, it has been conceptualised as an “interactive process of co-constructing knowledge” (Pohl et al. 2021, 22) that includes emotional and social factors (such as values) alongside cognitive factors (Boix Mansilla et al. 2016; Pohl et al. 2021). This type of interdisciplinary or transdisciplinary integration – depending on who is involved – is seen as a necessity for successful IDR/TDR and is often valued as their main epistemic goal and unique contribution (Defila and Di Giulio 2015; Huutoniemi and Rafols 2017). That is, because interdisciplinary/transdisciplinary integration is considered to account for complexity and thus to enable a deeper understanding of a problem and more comprehensive results and solutions (Defila and Di Giulio 2015). Integration therefore leads to novel insights and epistemic breakthroughs and consequently fosters technological innovation (Barry et al. 2008; O’Malley and Soyer 2012). Hence, it is widely used as a central indicator to assess IDR/TDR (Mansilla et al. 2006; Klein 2008).

In relation to integration, further values emerge: on the one hand, Huutoniemi & Rafols (2017) suggest *breadth* of the involved knowledge fields as a value to assess the quality of IDR, or to determine whether it would even qualify as such, as IDR is the combination of diverse intellectual contributions that will allow for novel insights through integration. On the other hand, *collaboration* stands out as a central value of IDR/TDR (Blanchard and Vanderlinden 2010; Polk 2015). Research in teams is considered to better address the complexity of the tackled problems, thanks to being able to combine breadth with disciplinary depth, represented by different experts in a collaboration, as well as due to increased creativity and ingenuity (Bozeman and Boardman 2014; Andersen 2016; Huutoniemi and Rafols 2017). It follows that it is the combination of integration, breadth and collaboration that allows IDR/TDR to make novel and valuable epistemic contributions. Breadth without integration, for example, might not create something new that goes beyond the sum of its parts (O’Rourke et al. 2016), and integration without collaboration might forgo the chance for aptly grappling with the complexities of IDR (Specht and Crowston 2022).

However, collaboration is considered to clash with two values central to the academic system: *autonomy* and *competition*. Firstly, aiming for integration through the means of collaboration makes researchers epistemically dependent on each other (Andersen 2016). When the result of integrative, collaborative interdisciplinary research is ‘more than the sum of its parts’ (Hoffmann et al. 2017), researchers need to rely on each other’s varying expertise. The same applies to the assessment of interdisciplinary contributions, where reviewers will build co-dependent opinions, trusting in others’ competencies (Andersen 2016). Secondly, valuing collaboration may stand in opposition to and, in practice, be hindered by valuing competition, which is considered essential for scholarly progress (Assimakopoulos et al. 2007; Hirsch and Luzadis 2013).

The value landscape around ‘research merit’ (Fig. 4) connects values that determine whether and why IDR/TDR are considered to be (or not to be) valuable as a form or research and knowledge production. It has revealed ‘repulsive’ values, normative expectations that are oftentimes perceived to be at odds with IDR/TDR, such as rigour, ‘scientificity’, or competition. At the same time, these connect to values that represent why IDR/TDR are considered to be an epistemically beneficial endeavour, namely breadth, integration, and collaboration. Framing these together under one value landscape highlights the pluralist normative expectations of IDR/TDR, especially given the diverse disciplinary contexts in which these values manifest with plural meanings.

## Discussion

This study seeks to help move the topic of values to the centre of attention in IDR/TDR practices and assessment. Our findings showed that IDR/TDR are deeply embedded in value landscapes that consequently shape their assessment. In what follows, we present two implications of our study: (i) the indeterminacy of certain values due to their embeddedness in particular value landscapes, and the importance of understanding the power dynamics at play, and (ii) the disconnect between different values in IDR/TDR and its effect on notions of accountability.

### Indeterminant values and power in research assessment

Our findings showed that the values that constitute the value landscapes of societal relevance and research merit are characterised by an indeterminacy caused by the plurality as well as the context-dependency of the values and their meanings. We illustrate this with a few examples. Presenting the value landscape of societal relevance, we have shown that the literature suggests that the exact meaning of ‘societal relevance’, i.e., what is the ‘thing’ that is valued when valuing relevance, depends on which and whose social values are considered in an IDR or TDR process (Lélé and Norgaard 2005). Here we consider it helpful to, once again, emphasise the perspective of values as enacted: in this case, the value of societal relevance only unravels its meaning once it is enacted through practices that for example, involve certain stakeholders and not others, or consider the needs of certain communities as pertinent, and those of others less. Moreover, as various and differing needs are taken into account, what is really ‘relevant’ might be plural. The same argument can be made for the value of legitimacy, which as an assessment criterion for TDR determines whether the research or its outcomes are “acceptable and trustworthy in the eyes of those who will use it” (Belcher et al. 2016, 12). Hence, legitimacy is dependent on the values and expectations of the prospective users or involved stakeholders, who might have diverse and opposing opinions of what is acceptable, thus introducing a plurality into it (Defila and Di Giulio 2024). Similarly, not only in the research practice, but also in the assessment practice of IDR/TDR, understandings of relevance or societal impact often differ, resulting in plural and co-existing ideas of what is to be assessed and thus valued in IDR/TDR (Derrick and Samuel 2016). The indeterminacy of societal relevance and related values, due to their plural and context-dependent meanings, defies the application of generalised standards in IDR/TDR assessment and contributes to its challenging nature (Belcher et al. 2020).

Additionally, we explored the value landscape that underlies notions of what makes IDR/TDR meritable as modes of research. It is, to an extent, characterised by a discourse that establishes the importance of certain values, such as scholarly quality or excellence, but also questions the ability of IDR/TDR to align with these. For IDR/TDR, the meanings of these values need to be translated across disciplines, each with its own epistemic values and indicators of quality and related expectations (Feller 2006; Boix Mansilla et al. 2016; de Sandes-Guimarães et al. 2022). The metaphor of value landscapes can offer a useful perspective by guiding attention to other values that impact contextual meanings of values like quality or rigour. For example, the quality of a simulation in computational sciences is measured by its increased simplicity while maintaining fit with empirically observed events. In contrast, cultural anthropology considers quality to consist in the richness and reflexivity of a ‘thick description’, rather than its simplicity (Geertz 1983; Hirsch Hadorn and Baumberger 2019). The context, in this perspective, includes the involved disciplines, which determine the plural values that become relevant for IDR/TDR in a specific setting. This has critical implications, as whether IDR/TDR are considered to be credible and legitimate will also impact whether they gain funding (Felt et al. 2016; König and Gorman 2017). This again is often decided via assessment procedures where experts with different interests and backgrounds first have to disentangle the plurality and context-dependency of the involved values. If this is not done, disagreements and uncertainty remain about what is ultimately valued, both among assessors and researchers, the latter of which are left insecure as to how they can successfully navigate the assessment of their IDR/TDR projects. The consequences are a lack of clarity and consensus on the type of data and indicators adequate for evaluating IDR/TDR, and a resulting lack of evidence about the effectiveness and value of these non-traditional research approaches that are currently observed (Ash 2019; de Rijcke et al. 2019; Adelle et al. 2020).

To grasp the significance of the indeterminacy of the values in IDR/TDR and its consequences, we need to acknowledge the powerful position of institutions conducting research assessment. In assessments, the “observed” is under the directive of the “scrutiniser’s” values (Shore and Wright 2000, 59), and assessing research is “a means of exercising control over knowledge” (Huutoniemi and Rafols 2017, 498). In making an assessment decision, assessors exercise a form of power by conducting a value judgement based on underlying values (Smit and Hessels 2021). Through this value judgement, they decide what is worth knowing and produce ‘matters of concern or care’, thus performing an act of valuation (Dussauge 2015). Thus, while a value like excellence might be embedded in assessment criteria, the way it is interpreted and applied determines the meaning the value takes on, illustrating how values are not stable entities, but unfold when enacted. This power generally lies with assessing institutions but may also be (unequally) distributed in inter- and transdisciplinary teams (Lélé and Norgaard 2005). Critically understanding questions of power in IDR/TDR is thus of utmost importance and requires further study (Fritz and Binder 2020), specifically from a perspective of values. As Dussauge (2015) argues, an analysis of values and their multiplicity creates an opportunity to understand processes of valuation at the core of power and politics.

We stress that we do not aim to call for a unification or standardisation of values in the assessment of IDR/TDR – contextuality and the resulting plurality of IDR/TDR processes, outcomes and impacts are central to ensure their societal relevance. Rather, we see the need to study and better understand values at play in IDR/TDR and their assessment to support critical reflection on and disentangle normative expectations and related practices, which will consequently lead to clearer and more adequate assessment criteria and procedures for IDR/TDR. A first step in this direction is to acknowledge that multiple values coexist, and that their balance depends on context and interpretation. For this, it helps, we argue, to understand values not as isolated, but as embedded in value landscapes, thus always influencing and being influenced by other values, as well as enacted through value practices such as research assessment procedures.

### Connecting disconnected notions of accountability

In addition to the indeterminant nature of the values in IDR/TDR, the value landscapes are further characterised by a disconnect. We use this term to summarise the tensions and fragmentations between and within the value landscapes, illustrating the difficulty of bringing them and related expectations into accord in productive and integrative ways.

In assessment processes, IDR/TDR are often subject to metric indicators like impact factors, rankings, and number of publications, which transport expectations of scientificity and values like rigour and excellence that represent disciplinary conceptualisations of success (Fochler and Rijcke 2017). At the same time, funders themselves increasingly embed their programmes in the value landscape of societal relevance to push for problem-oriented research (Bandola-Gill 2019). As a result, IDR/TDR are under pressure to produce research that contributes to practical solutions to societal challenges while still being subject to expectations of epistemic novelty and research excellence (Dupin et al. n.d; Maasen and Lieven 2006; Henze 2021; Laursen et al. 2022). However, as support for IDR/TDR’s orientation towards societal relevance coexist with expectations of excellence and rigour, in practical assessment processes, these are perceived to stand in tension and require trade-offs (Derrick and Samuel 2016; Bandola-Gill 2019). Assessment infrastructures, while aiming to ensure contributions to both scholarly and societal interests, have not yet been adequately adapted to reconcile the two due to the existing tensions (Bandola-Gill 2019).

Flinders (2013) conceived of demands for societal relevance of scientific research – in this case, of social sciences and specifically political sciences – as ‘external’ pressures, imposed on researchers by the ‘public’ through funding mechanisms, disconnected from the aspirations for high-quality or excellent research, which he characterised as ‘internal’, coming from and belonging to academic culture. Based on our findings, we argue that both demands have become central to the ‘internal’ governance of IDR/TDR, but remain fragmented. While expectations of societal relevance and research merit increasingly blur and co-occur in this context, the tensions between them lead to a disconnect between the two value landscapes and inhibit a productive way of connecting and balancing the two. A consequence of that is the negotiations in assessment panels that Derrick & Samuel (2016) describe about whether and how to prioritise societal impact over research quality. These opposing expectations and subsequent negotiations are also ingrained in many definitions of IDR/TDR. Although IDR/TDR are generally understood as research modes, most discourses denote a direct connection to societal demands (Vienni-Baptista et al. 2022).

These fragmented, disconnected values and expectations need to be reconciled in order for the value of IDR/TDR processes, outcomes and inputs to be aptly captured and adequately assessed. Moreover, strategies need to be developed to honour the plurality and contextuality of the values in IDR/TDR, not only in research, but also in assessment, despite the necessity for definitive value judgements in the latter. Similar concerns and demands have been discussed under the notion of accountability in IDR/TDR. Accountability is generally a major goal of and thus justification for conducting research assessment (Strathern 2000; Chelimsky 2006). ‘Rituals of verification’ (Power 1999) in the form of assessment procedures aim at verifying the quality of a scrutinised unit in order to establish accountability towards a designated audience (Shore and Wright 2000; Strathern 2000). Traditionally, the accountability commitments of academic research, including IDR/TDR, have been considered to be towards the ‘scientific method’ and their achievement has been measured by disciplinary standards (Huutoniemi 2016; Horcea-Milcu et al. 2024). It has been argued, however, and our findings confirmed, that IDR/TDR respond to more diverse accountability demands, including that of more practically contributing to societal transitions in the light of pervasive challenges (Horcea-Milcu et al. 2024). This is supported by the “logic of accountability”, presented by Barry, Born, and Weszkalnys (2008) as a primary justification for interdisciplinarity, as accountability of research towards society is considered to be increased due to high societal relevance achieved through contextualisation and alignment with societal values (Huutoniemi 2014; Frickel et al. 2017; Henze 2021). Thus, while the original setting of scientific knowledge production rested on the promise of fundamental, reliable knowledge in return for the provision of public funds, institutions nowadays increasingly press for research providing societally relevant impact to increase accountability in the light of public funding (Gibbons 1999; Nowotny 2016). It is the practices of IDR/TDR that establish their accountability – arguably both in epistemic and in societal ways (Huutoniemi 2016) – and therefore the enactment of underlying values that makes them accountable. Thus, in addition to considering towards whom and what IDR/TDR ought to be accountable (Huutoniemi 2016), we suggest taking into account what this implies for disentangling, balancing, and reconciling different values in IDR/TDR practice and assessment to ensure the assessment serves its purpose in establishing accountability aligned with the context.

Strategies to unravel and embrace the plurality and context-dependent meanings of values involved in assessment, but also to reconcile and balance expectations of societal relevance and research merit, will support efforts to develop adequate assessment to ensure IDR’s and TDR’s accountability by both considering their scholarly quality and societal impact. Such efforts have become of essential concern in recent years^4^ (Smit and Hessels 2021). Institutions involved in research assessment may contribute to alleviating the indeterminacy and the disconnect by dealing with pluralistic value landscapes through reflection about values, normative assumptions and decisions (Fazey et al. 2018). Thus, we argue, institutions establish accountability both in terms of a knowledge production process that meets scholarly and societal expectations, as well as in terms of an accountable assessment process with an awareness of present power dynamics. Ideally, such reflective processes are followed by measures of transparency to make otherwise hidden aspects of research available for discussion and critique (Robinson et al. 2016; Brigstocke et al. 2017). Institutions involved in assessment can learn from IDR/TDR practices themselves, where the inclusion of societal values and values of stakeholders by default creates a form of accountability that matches the research’s purpose and alleviates power imbalances (Maasen and Lieven 2006; Klein 2014). In IDR/TDR practices, reflection on and clarification of the values involved in the research process allow to examine their effects and are thus considered to make the research process more objective (Robinson et al. 2016). Explicitly negotiating values in the assessment of IDR/TDR, and grounding them in local value landscapes of researchers and their stakeholders, could lead to developing new indicators and definitions of relevance and impact that reconcile the two value landscapes sketched in this article and redistribute the power to explicitly decide which values gain normative force (Belcher et al. 2017; Brigstocke et al. 2017; Henze 2021).

Our study has shown that the metaphor of value landscapes is a useful conceptualisation to characterise the relationships between the values, illustrating how they stand in relation, influencing each other’s meaning. While Schulz et al. (2017) characterised those relationships as hierarchies, our findings show the need to conceptualise them as dependencies, creating dynamic value orders in which the enactment of some values (e.g., societal change) are a prerequisite for other values (e.g., robustness and usability of the knowledge) and creates tensions with or are perceived to be disconnected from other values (e.g. rigour or excellence). We have used a dichotomous structure to present two distinctive value landscapes and their differing relations to IDR/TDR practice and assessment, but we want to highlight that these relationships and interdependencies exist *across* the value landscapes. The boundaries of these value landscapes increasingly blur and accompany a shift in the meaning of certain values like quality, robustness, legitimacy or reliability. These might once have had purely scientific connotations, but are now increasingly applied in a societal, problem-oriented context (see e.g. Gibbons 1999). This blurring, representing the shift from valuing purely epistemic contributions to valuing contributions towards societal change and impact, which has taken place in research funding and governance, further implies that the distinction between epistemic and non-epistemic values, i.e., values belonging to the scientific domain and other values belonging purely to the societal domain, becomes even less feasible. IDR/TDR are required to fulfil diverse expectations and are embedded in plural and overlapping value landscapes, as our findings demonstrate. Not only do they break out of the realm of science and extend into the “real world,” and thereby blurring the distinction between the two (Maasen and Lieven 2006; Bandola-Gill 2019), but the success and value of IDR/TDR are also dependent on their consideration of non-epistemic values. Thus, along the two landscapes, we illustrate how ‘values’ serve as a useful sensitising concept (Bowen 2006) for finding new ways of studying, understanding, and theorising the entanglement of IDR/TDR and research assessment, and to better understand the barriers resulting from the latter to the former.

In this study, we analysed value landscapes relevant to both IDR and TDR to improve understanding of emerging barriers at the intersection of research practices and research assessment across these two modes of research. Given this scope and the corresponding selection of literature, the study provides only limited insight into the nuances of differences between IDR and TDR with respect to values. Addressing these differences will require further empirical research.

## Conclusion

We identified two blurring and overlapping value landscapes in which IDR/TDR practice and assessment are embedded. Together, they allow us to gain a comprehensive understanding of some of the intersections between IDR/TDR practice and their assessment. The indeterminacy of the values in the two value landscapes and the disconnect that characterises their relationships reveal the need to rethink power and accountability in IDR/TDR assessment. This would allow reappropriation and alignment of values like quality and relevance with the capacities and potential of collaborative research (Shore and Wright 2000). We advocate that an elevated understanding of values can support the disentanglement and reconciliation of indeterminant and disconnected expectations, which IDR/TDR must live up to and which may create adverse consequences when transported through assessment mechanisms. It is necessary to establish a culture of transparent and collaborative consideration of these values to alleviate these barriers. A transparent reflection on values among researchers and institutions involved in assessment would not only shed light on but also allow rearrangement of the power distribution in IDR/TDR practice and assessment and offer a base for discussion of goals, purpose, and related accountabilities.

## Supplementary information

Supplementary information

## Data Availability

The dataset underpinning this literature review, together with the search queries used to retrieve the publications and an accompanying document detailing the review process, has been deposited and is publicly accessible in the ETH Research Collection. The dataset is available at 10.3929/ethz-b-000632756 and the search strings and accompanying documentation are available at 10.3929/ethz-b-000632748.

## Footnotes

1 Funded by the Swiss National Science Foundation (PRIMA No. 201582). More details on: https://csts.ethz.ch/research/intersections.

2 Databases and strings are Open Access (Schaltegger and Vienni-Baptista 2023; Schaltegger et al. 2023).

3 Examples of such efforts to reform research assessment are the Quality & Rigour Project of the Toolkits & Methods Working Group of the ITD Alliance, or the working group of societal impacts in the Coalition for Advancing Research Assessment (CoARA).

4 Throughout the Findings section, values that are represented in Figs. 3 and 4 are italicised the first time they are mentioned.
