# Supplementary material for: Value landscapes in interdisciplinary and transdisciplinary research and assessment: exploring indeterminacies and disconnects
Source: Humanit Soc Sci Commun. 2026 Mar 10;13(1):407. doi: 10.1057/s41599-026-06785-0 (PMC13053187; doi:10.1057/s41599-026-06785-0)
Supplement: Supplementary file 1 — Supplementary information [file 41599_2026_6785_MOESM1_ESM.pdf]

Inclusion and exclusion criteria guiding the literature search and selection

	Selection criteria	Reason for criteria
**Scope**	Questions for the Literature Review are derived from the research questions.<br>- Q1: How are ID and TD understood and conceptualised in research and in policy?<br>- Q2: What are suitable theories and methods to investigate inter- and transdisciplinary knowledge production processes?<br>- Q3: What are the specific challenges to interdisciplinary and transdisciplinary research (practices, cultures, spaces) and to its responsiveness to scientific and societal demands?<br>- Q4: What are the intersections that exist between practices, cultures and policy in ID/TD and how do they enable impact on scientific and societal needs?	Relationship to research questions.
**Topic coverage**	All papers that contain interdisciplinar* or transdisciplinar*	Focus of our research project is on inter- and transdisciplinarity.
**Exclusion criteria**	• Publications that exclusively relate to teaching and learning.	• Publications that serve as a resource for educators or students respectively to teach or learn about inter-/transdisciplinarity, or that serve as guidance on how to develop an inter-/transdisciplinary research design.<br>• Example: Menken, S., Keestra, M., Rutting, L., Post, G., de Roo, M., Blad, S., & de Greef, L. (2016). An introduction to interdisciplinary research: Theory and practice.
	• Publications whose title or abstract is not in English or German.	• Relevant publications from Switzerland will be available in English or in one or several national languages, German being the most dominant one for the context in which the project is developed.
	• Papers that analyse the academic/professional trajectory of an individual person.	• The focus of our project is on collective and collaborative forms of inter- and transdisciplinary research.

	Selection criteria	Reason for criteria
		Individual trajectories fall outside this scope.
	• Publications about inter- or transdisciplinary collaborations that do not relate to research activities and/or policy.	• Publications that, for example, deal with interdisciplinary collaboration in a private sector company, with no relation to research activities or policy.<br>• Example: Huth, D., Vilser, M., Bondel, G., & Matthes, F. (2020). Empirical Task Analysis of Data Protection Management and Its Collaboration with Enterprise Architecture Management. In ICEIS (2) (pp. 656-665).
	• Book reviews.	• In this literature review, we concentrate on academic research publications. We don’t consider book reviews research publications and therefore exclude them.
	• Interviews.	• We don’t consider interviews to be academic literature and hence don’t include interviews. This does not concern academic publications that use interviews as data or method.
	• Bachelor, Master, and PhD theses.	• These publications are not usually included in the databases available for the project.

	Selection criteria	Reason for criteria
**Inclusion criteria**	The documents must be of one of the following types:<br>- Journal articles published in a peer-review process<br>- Books or book chapters written or edited by people with a research or academic affiliation<br>- Written outputs of conferences targeting researchers or people with an academic affiliation<br>and meet at least one of the following criteria:	• Exhaustive list of the document types we consider to be academic literature.
	• Analyse and/or evaluate collaborative knowledge production processes and/or inter-/transdisciplinary case studies empirically (quantitatively and/or qualitatively).	• Explorations of inter-/transdisciplinary research can inform all four questions.<br>• Example: Cuppens, A., Smets, I., & Wyseure, G. (2013). Identifying sustainable rehabilitation strategies for urban wastewater systems: A retrospective and interdisciplinary approach. Case study of Coronel Oviedo, Paraguay. *Journal of Environmental Management*, *114*, 423-432.
	• Present best practices of or recommendations for inter-/transdisciplinary research and/or integration processes.	• Recommendations or presentations can inform all four questions.<br>• Example: Lang, D. J., Wiek, A., Bergmann, M., Stauffacher, M., Martens, P., Moll, P., ... & Thomas, C. J. (2012). Transdisciplinary research in sustainability science: practice, principles, and challenges. Sustainability science, 7(1), 25-43.
	• Outline concepts/definitions and/or understandings of inter-/transdisciplinarity or synonymous concepts as the main focus of the paper (collab*, co-prod*, interdisciplinar*, transdisciplinar*, participa*, cooperat*).	• Relates to Q1.<br>• Example: Barry, A., Born, G., & Weszkalnys, G. (2008). Logics of interdisciplinarity. *Economy and society*, *37*(1), 20-49.
	• Explore methods and/or theories to investigate and/or evaluate inter-/transdisciplinary knowledge production processes.	• Relates to Q2.<br>• Example: Callard, F., Fitzgerald, D., & Woods, A. (2015). Interdisciplinary collaboration in action: tracking the signal, tracing the

	Selection criteria	Reason for criteria
		noise. Palgrave communications, 1(1), 1-7.
	• Explore factors that challenge, hinder or enable inter- and transdisciplinary research (condition*, indicat*).	• Relates to Q3.<br>• Example: Engwall, L. (2018). Structural conditions for interdisciplinarity. European Review, 26(S2), S30-S40.
	• Explore and/or evaluate the impact of inter-/transdisciplinary research on societal, scientific and/or policy domains.	• Relates to Q3.<br>• Example: Hadorn, G. H., Bradley, D., Pohl, C., Rist, S., & Wiesmann, U. (2006). Implications of transdisciplinarity for sustainability research. Ecological economics, 60(1), 119-128.
	• Explore how policy, research, culture and society mutually affect each other and discuss the intersections that emerge from collaborative interactions of these domains.<br>• Explore how inter-/transdisciplinarity and research policy shape each other.	• Relates to Q4.<br>• Example: Sapat, A. (2021). Lost in translation? Integrating interdisciplinary disaster research with policy praxis. Risk analysis, 41(7), 1232-1239.
**Geographic barriers**	No geographic barriers.<br>Balance between countries represented in the analysed corpus was pursued.	
**Language**	English	
**Period of time**	2000 - 2022	
**Source**	Scopus, Web of Science, JSTOR, Scimago	
